# Differentiation in TCM patterns of chronic obstructive pulmonary disease by comprehensive metabolomic and lipidomic characterization

**DOI:** 10.3389/fimmu.2023.1208480

**Published:** 2023-07-10

**Authors:** Jiansheng Li, Xinguang Liu, Yanmin Shi, Yang Xie, Jianya Yang, Yan Du, Ang Zhang, Jinyan Wu

**Affiliations:** ^1^ Co-construction Collaborative Innovation Center for Chinese Medicine and Respiratory Diseases by Henan & Education Ministry of P.R. China, Henan University of Chinese Medicine, Zhengzhou, China; ^2^ Henan Key Laboratory of Chinese Medicine for Respiratory Disease, Henan University of Chinese Medicine, Zhengzhou, China; ^3^ Academy of Chinese Medical Sciences, Henan University of Chinese Medicine, Zhengzhou, China; ^4^ Department of Respiratory Diseases, The First Affiliated Hospital of Henan University of Chinese Medicine, Zhengzhou, China

**Keywords:** biomarker, COPD, lipidomics, metabolites, traditional Chinese medicine pattern

## Abstract

**Introduction:**

Chronic obstructive pulmonary disease (COPD) is a complex disease involving inflammation, cell senescence, and autoimmunity. Dialectical treatment for COPD with traditional Chinese medicine (TCM) has the advantage of fewer side effects, more effective suppression of inflammation, and improved immune function. However, the biological base of TCM pattern differentiation in COPD remains unclear.

**Methods:**

Liquid Chromatography-Quadrupole-Orbitrap mass spectrometry (LC-Q-Orbitrap MS/MS) based metabolomics and lipidomics were used to analyze the serum samples from COPD patients of three TCM patterns in Lung Qi Deficiency (n=65), Lung-Kidney Qi Deficiency (n=54), Lung-Spleen Qi Deficiency (n=52), and healthy subjects (n=41). Three cross-comparisons were performed to characterize metabolic markers for different TCM patterns of COPD vs healthy subjects.

**Results:**

We identified 28, 8, and 16 metabolites with differential abundance between three TCM patterns of COPD vs healthy subjects, respectively, the metabolic markers included cortisol, hypoxanthine, fatty acids, alkyl-/alkenyl-substituted phosphatidylethanolamine, and phosphatidylcholine, etc. Three panels of metabolic biomarkers specific to the above three TCM patterns yielded areas under the receiver operating characteristic curve of 0.992, 0.881, and 0.928, respectively, with sensitivity of 97.1%, 88.6%, and 91.4%, respectively, and specificity of 96.4%, 81.8%, and 83.9%, respectively.

**Discussion:**

Combining metabolomics and lipidomics can more comprehensively and accurately trace metabolic markers. As a result, the differences in metabolism were proven to underlie different TCM patterns of COPD, which provided evidence to aid our understanding of the biological basis of dialectical treatment, and can also serve as biomarkers for more accurate diagnosis.

## Introduction

Chronic obstructive pulmonary disease (COPD) is characterized by continuous airflow restriction, which is related to the enhanced chronic inflammatory response of the airway and lungs to toxic particles or air. It is estimated that COPD-related mortality will reach 1 billion by the end of the 21st century (1). The pathological process of COPD is closely related to immune and inflammatory processes. Smoking, exposure to biomass fuel, dust, household fumes and pesticides, tuberculosis infection, occupational exposure, and frequent childhood infections are considered to be the main causes of COPD. These factors can cause inflammation-related processes including cells and mediators of both initiative and adaptive immunity and reactive oxygen species, which lead to lung tissue damage and loss. Meanwhile, recent studies have shown that autoimmunity also plays a role in COPD. The autoimmune mechanism explains the sustained unregulated inflammatory process after quitting smoking, as the initial inflammation and environmental damage in the lungs expose certain epitopes of autoimmune attack (2). At present, the treatment strategy for COPD can only control respiratory symptoms but cannot improve damaged lung function to reduce the occurrence of acute exacerbation.

Recently, the dialectical treatment of COPD with traditional Chinese medicine (TCM) has demonstrated advantages including fewer side effects and more effective promotion of lung function recovery (3). Dialectical treatment is recognized as an ancient precision medicine; that is, patients with the same disease can be divided into different “TCM patterns”. The TCM pattern is characterized by a certain cause, location, nature, and developmental tendency of a disease at a specific stage and is identified through a comprehensive analysis of the clinical symptoms and signs gathered by a practitioner using inspection, auscultation, olfaction, interrogation, and palpation of the pulses. Based on TCM pattern differentiation, different treatment strategies were given to patients. The stable stage of COPD can be classified into three common TCM patterns: Lung Qi Deficiency, Lung-Kidney Qi Deficiency, and Lung-Spleen Qi Deficiency. In a four-center, open-label randomized controlled study involving a total of 352 patients, TCM dialectical treatment was shown to have beneficial effects on measured outcomes in COPD patients over the 6-month treatment and 12-month follow-up period, with no relevant between-group differences in adverse events (4). In model rats with COPD, the formula used in the dialectical treatment for COPD has also been widely proven to reduce inflammation and improve immune function (5–8). However, the biological basis of TCM patterns remains unclear; thus, the diagnosis of TCM patterns still mainly depends on TCM practitioners’ subjective judgment, and there is no quantitative standard yet to help them differentiate TCM patterns and then select specific treatment strategies.

Previously, the biological basis of TCM patterns was commonly elucidated by low-throughput molecular biological methods. However, this is insufficient due to the complexity of TCM patterns. With the advent of systems biology, high-throughput, high-coverage genomics, transcriptomics, proteomics, and metabolomics methods, combined with robust bioinformatics and computational tools have been widely and effectively applied in the biological basis research of TCM patterns. Among them, metabolomics is the most commonly used method to study the biological basis of TCM patterns because of its more popular technology and equipment, easier operation, and lower cost. Wu et al. used a strategy that integrated proteomics, metabolomics study for clinic samples, and network pharmacology for TCM pattern differentiation of two typical syndromes of coronary heart disease: Cold Congealing and Qi Stagnation, and Qi Stagnation and Blood Stasis (9). Ye et al. used a urine and serum metabolomics study for the pattern differentiation of gastroesophageal reflux disease (10).

For COPD, metabolism disturbance is found as a significant pathological feature and is closely related to inflammation and immunity. For example, compared to healthy subjects, COPD patients were found to have increased levels of betaine and choline (1) and an increased ratio of carnitine to acylcarnitine (11). In addition, altered from the stable stage to acute exacerbation stage of COPD was accompanied by change of trihexosylceramide level (12), sphingolipids, ether-containing glycerophospholipids, phosphatidylglycerols, and glycerol lipids. Among them, the reduction of plasmalogen content is related to peroxisomal dysfunction, oxidative stress, or phospholipase activation; the reduction of phosphatidylglycerol (PG) is related to the damage of pulmonary surfactant; and the increase in ceramides is related to pulmonary vascular cell apoptosis (13). Therefore, deciphering the biological basis and diagnostic biomarkers of different TCM patterns of COPD based on metabolic disturbance may be an effective strategy.

Mass spectrometry (MS) coupled with ultra-high performance liquid chromatography (UHPLC)-based metabolomics and lipidomics are the predominant strategies for comprehensive profiling metabolism in a biosystem and have been applied to the biomarker screening of many diseases. In the present study, UHPLC-Q-Exactive Orbitrap-MS-based metabolomic and lipidomic analysis was used to identify the biological differences between the different TCM patterns of COPD at the metabolic level. As a result, a biomarker panel that characterizes three TCM patterns of COPD was detected, which may enhance our understanding of the scientific connotations of dialectical treatment for COPD and may also be used to improve the diagnosis accuracy of different patterns of COPD and, further, the personalized management.

## Materials and methods

### Chemicals and materials

Ammonium acetate, LC-MS-grade formic acid (FA), isopropanol and acetonitrile, and HPLC-grade methanol and methyl tert-butyl ether (MTBE) were purchased from Fisher Scientific (Geel, Belgium). Lipidomics Isotope-labelled internal standards (13) and Lyso-phosphatidylcholine (LPC) 19:0 (for metabolomics analysis) were purchased from Avanti Polar Lipids (Alabaster, AL, USA). Isotope-labeled internal standards for metabolomics analysis, including tryptophan-d5, valine-d8, phenylalanine-d5, palmitic acid-d3, cholic acid-d4, stearic acid-d3, carnitine C8:0-d3, and carnitine C16:0-d3, were obtained from Cambridge Isotope Laboratories (Tewksbury, MA, USA). Ultrapure water was prepared using the Milli-Q system (Millipore, Billerica, USA).

### Subjects and study design

A total of 212 subjects were retrospectively enrolled in this study from December 2019 to June 2021 at The First Affiliated Hospital of Henan University of Chinese Medicine, including 41 healthy subjects and 171 patients with COPD (Global Initiative for Chronic Obstructive Lung Disease stage I/II or A/B). Patients with COPD were classified into three TCM patterns, including Lung Qi Deficiency (n=65), Lung-Kidney Qi Deficiency (n=52), and Lung-Spleen Qi Deficiency (n=54), which were diagnosed as described in our previous work (4, 14), according to the Diagnostic Criteria of TCM Syndromes of Chronic Obstructive Pulmonary Disease (updated in 2011) (3, 15):

#### Pattern 1

Lung Qi Deficiency, is characterized as panting and coughing and shortness of breath, which are worse when active; lassitude and spontaneous sweating; being prone to catching a cold; a pale tongue with white fur; and a deep thready pulse or a thready weak pulse.

#### Pattern 2

Lung-Kidney Qi Deficiency, is characterized as panting and shortness of breath, which are worse when active; lassitude and spontaneous sweating, which are worse when active; being prone to catching a cold; weakness in the lower back and knees; tinnitus, vertigo, or asthenia facial edema; profuse urination, frequent urination at night, or urine released with coughing; a pale tongue with white fur; and a deep thready pulse or a thready weak pulse.

#### Pattern 3

Lung-Spleen Qi Deficiency, is characterized as coughing and shortness of breath, which are worse when active; lassitude and spontaneous sweating; being prone to catching a cold; poor appetite or eating less; bloating in the gastric cavity, abdominal distension or loose stools; enlarged tongue with white or greasy fur; and a deep thready pulse, a deep slow pulse, or a thready weak pulse.

Patients with asthma, bronchiectasis, pleural effusion, founder’s pneumoconiosis, silicosis, asbestosis, or other diseases that could be confused with COPD were excluded. The baseline characteristics and clinical parameters of the study population are presented in **Table 1**. A fasting blood sample was drawn by venipuncture between 07:00 and 09:00 h and allowed to stand at room temperature for ≥60 min before centrifugation at 3000×*g* for 10 min at room temperature, then stored at −80°C until use. This study was conducted in accordance with the Declaration of Helsinki and was approved by the ethics committee of The First Affiliated Hospital of Henan University of Chinese Medicine (Ethical approval number 2019HL-016-02). All participants provided written informed consent.

**Table 1:** Baseline characteristics and clinical parameters of the study population.

	Healthy subjects (n=41)	Lung Qi Deficiency COPD (n=65)	Lung-kidney Qi Deficiency COPD (n=52)	Lung-spleen Qi Deficiency COPD (n=54)	P-value
Age, years	55 (50-65)	61 (42-75)	64 (45-74)	64 (44-74)	<0.001
Sex, % male	25 (60.98%)	50 (76.92%)	40 (76.92%)	43 (79.63%)	0.192
Body mass index	24.76 (14.69-31.55)	24.61 (17.83-31.25)	23.58 (16.16-30.92)	23.46 (17.72-29.99)	0.698
Current smoker, %	15 (36.59%)	38 (58.46%)	26 (50%)	32 (59.26%)	0.104
Course of COPD, months	0	48 (1-516)	84 (6-360)	60 (1-245)	<0.001
FVC(L)	3.61 (2.15-5.43)	2.61 (0.91-3.42)	2.2 (0.71-5.28)	2.4 (1.11-4.32)	<0.001/<0.001*
FEV1(L)	2.61 (1.96-3.84)	1.58 (0.5-2.19)	1.4 (0.79-3.07)	1.33 (0.47-2.87)	<0.001/0.034*
FEV1/FVC (%)	78.92 (70.71-91.16)	60.61 (21.07-216.08)	50.00 (27.01-197.18)	25.24 (17-75.46)	<0.001/0.015*

Baseline characteristics were expressed as median (interquartile range) or n (%).

P<0.05 was considered statistically significant.

†P-values were calculated by the ANOVA test for continuous variables or the chi-squared test for categorial variables.

* P-values of ANOVA test between the groups of three COPD TCM patterns.

### Metabolomic analysis

Serum samples were thawed at 4°C. Then 480 μL ACN that contained metabolite ISs with the concentration listed in **Table S1** was added to 120 μL serum to remove the protein. After vortexing for 1 min and standing for 15 min, the mixture was centrifuged at 18 000 g for 15 min at 4°C. Then two aliquots (250 μL for each) of the supernatant were taken and dried in a vacuum centrifuge. The residues were reconstituted in 50 μL 20% ACN before analysis. Quality-control (QC) samples were obtained by mixing 10 μL of each serum sample and prepared as the real samples. In the analytical sequence, one QC was regularly inserted after six real samples to monitor the stability of the system.

A Dionex Ultimate 3000 Ultra Performance Liquid Chromatography coupled to a Q-Exactive Mass Spectrometry (Thermo Fisher Scientific, Bremen, Germany) was used for metabolomics analysis. In positive mode, an ACQUITY BEH C18 column (2.1 mm × 100 mm, 1.7 µm) (Waters, Milford, MA, USA) was employed at a column temperature of 30°C. 0.1% FA in H_2_O and 0.1% FA in ACN were used as mobile phases A and B at a flow rate of 0.35 mL/min. The LC gradient was as follows: it started as 5% B for 1 min, then increased to 100% B in 24 min, followed by a 4 min wash at 100% B and a 5 min re-equilibration at 5% B. In negative mode, an ACQUITY HSS T3 column (2.1 mm × 100 mm, 1.8 µm) (Waters, Milford, MA, USA) was employed at a column temperature of 40°C. 5 mM ammonium acetate in water and methanol were used as mobile phases C and D, at a flow rate of 0.35 mL/min. The LC gradient was as follows: it started as 100% C for 1 min, then increased to 95% D in 19 min, followed by a 5 min wash at 95% D and a 5 min re-equilibration at 100% C. The injection volume was 2 μL.

Mass spectrometry detection was conducted respectively in positive and negative modes with the spray voltage at 3.5 and 2.8 kV. For both modes, the flow rates of the sheath gas and auxiliary gas were set at 40 and 10 arbitrary units, and the capillary temperature was 325°C. For sequence analysis, full MS mode was employed with the scan range from 70 to 1000 m/z and the resolution at 70 000. For identification analysis, full MS-ddMS2 mode was employed, and the parameter was set as follows: resolution of MS and MS2, 70 000 and 17 500; Top N, 5; dynamic exclusion time, 6 s; normalized collision energy, 20, 40, and 60.

Raw data files were processed by Compound Discoverer 2.1 (Thermo Fisher Scientific) with untargeted workflow, including retention time alignment, compound detection, background annotation, formula prediction, and database search. Then the exported areas of each compound in the real samples were calibrated by one metabolite standard, which was selected based on the performance of metabolite standards (with the minimum RSD) in the QC samples (16, 17). After calibration, compounds with RSD greater than 30% in QC samples were removed.

Multivariate analysis with SIMCA 14.0 (Umetrics, Malmo, Sweden) and univariate analysis with SPSS Statistics 19.0 (IBM, Armonk, NY, USA) were sequentially performed to screen differential metabolites. Partial least squares discriminant analysis (PLS-DA) with unit variance scaling was performed to maximize the differences between the healthy control group and the COPD group and distinguish the important metabolites contributing to the classification. Compounds with variable importance in the projection (VIP) values > 1.0, p-values of Student’s t-test < 0.05, and fold change > 1.3 or < 0.77 were considered significantly changed.

As to the identification of differential metabolites, those with candidates from the mzCloud database in the search results of Compound Discoverer 2.1 were checked manually. Both the deviation of the precursor ions and the match of the MS/MS spectrums were taken into consideration. For differential metabolites with similarity matching with carnitine or LPC, the MS/MS spectrums were checked for the characterized product ion of 85.0291 or 184.0733 for carnitine or LPC, then the precursor ions were searched against The Human Metabolome Database (HMDB) 5.0 (https://www.hmdb.ca) and LipidMaps (https://www.lipidmaps.org) with mass tolerance set at 10 ppm to determine the structures.

### Lipidomics analysis

Samples were analyzed using the same LC-MS system as the metabolomics study. A reversed-phase ethylene bridged hybrid C8 column (2.1×100 mm, 1.7 µm; Waters, Milford, MA, USA) was used for the chromatographic separation of lipids; The chromatographic and MS conditions and data process method have been described previously (13).

## Results

### Patient demographics and clinical outcomes

As presented in **Table 1**, the ages of 171 COPD patients are between 42 and 75. As expected, there were more male patients than female patients (76.9-79.63% male) with COPD, and the patients had a high smoking rate (50-59.26%); smoking affects lung function in many ways and has been confirmed as one of the causes of COPD. At the same time, the phenotypes caused by smoking will also be significantly different from other factors (18). Evidence has shown that, compared to smokers, never smokers with chronic airflow limitation have fewer symptoms, milder disease, a lower burden of systemic inflammation, and a lower risk of lung cancer or cardiovascular comorbidities but a higher risk of pneumonia and mortality from respiratory failure. Therefore, in the grouping, we ensured that there was no significant difference between healthy subjects and COPD patients of different TCM patterns in sex ratio, body mass index (BMI), and smoking ratio. Pulmonary function is the main clinical standard for identifying COPD. Compared with healthy subjects, the forced vital capacity (FVC, L), forced expiratory volume in one second (FEV1, L), and FEV1/FVC (%) of patients in COPD groups were significantly reduced. For FVC (L), Lung-Kidney Qi Deficiency < Lung-Spleen Qi Deficiency < Lung Qi Deficiency, for FEV1 (L), Lung-Spleen Qi Deficiency < Lung-Kidney Qi Deficiency < Lung Qi Deficiency. For the course of disease, Lung-Kidney Qi Deficiency> Lung-Spleen Qi Deficiency > Lung Qi Deficiency, which ranged from 1 to 516 months. Therefore, in general, the course of Lung Qi Deficiency patients is shorter, and the decrease in lung function is less than the other two types of TCM patterns.

### Cross-comparisons of patients with COPD of different patterns by metabolomics

**Figure 1:**
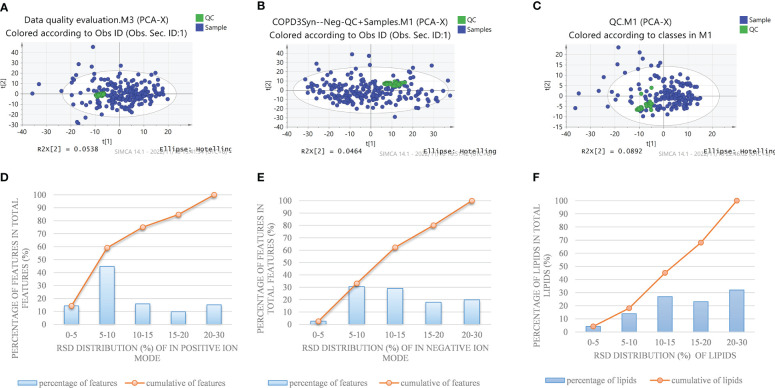
PCA plot of detected features in a QC sample and test sample by nontargeted metabolomics methods in the positive **(A)** and negative **(B)** ion modes, and **(C)** pseudotargeted lipidomics. Reproducibility of commonly detected features in the positive **(D)** and negative **(E)** ion modes and **(F)** pseudotargeted lipidomics.

In order to systematically illustrate the metabolic intervention of COPD patients with different TCM patterns, UHPLC-Q-Orbitrap MS was used to analyze the serum samples in both negative and positive ion modes. The representative total ion current of serum samples from the healthy subjects, COPD of Lung Qi Deficiency, Lung-Kidney Qi Deficiency, and Lung-Spleen Qi Deficiency was obtained under optimal conditions. **Figure 1** shows the stability test results of metabolomics and lipidomics. Firstly, PCA analysis was employed to show the variation of features in QC samples. **Figures 1A**, **B** showed the snapshots of test samples and QC samples measured in positive and negative ion modes, respectively. It can be seen that QC samples were mostly concentrated in the scatter diagram of PCA, which proves that the method was relatively stable during the experimental process. At the same time, **Figures 1D**, **E** show the relative error of the features detected in the positive and negative ion modes in the QC samples. It can be seen that the RSD of most of the features detected is within 5-15%, which also indicates the good performance of the analytical method.

**Figure 2:**
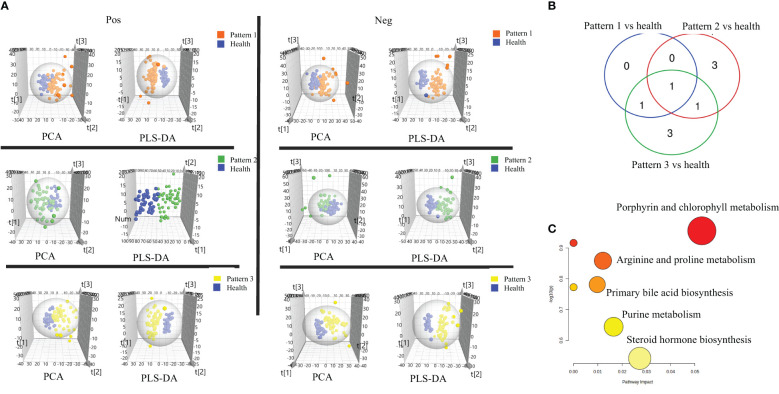
**(A)** Score plots of PCA and PLS-DA based on the cross-comparison of Lung Qi Deficiency (Pattern 1) vs health, Lung-Kidney Qi Deficiency (Pattern 2) vs health, and Lung-Spleen Qi Deficiency (Pattern 3) vs health. **(B)** Venn diagrams and **(C)** Biopathway enrichment of the changed metabolites for COPD patients with 3 TCM patterns vs healthy subjects by metabolomic analysis.

In negative ion mode, 2234 features were identified and used for the subsequent univariate and multivariate analyses. Firstly, PCA analysis was employed to give snapshots of alterations in metabolic profiling introduced by different TCM patterns of COPD. As presented in **Figure 2A**, there were well-demarcated trends between samples of 3 TCM patterns of COPD and healthy subjects. Then, PLSDA analysis was used to filter the changed metabolites. Similarly, the differences between COPD with three TCM patterns and healthy subjects were all notable in the PLS-DA 3D scatterplot, with a cumulative R2Y of 0.986 and Q2 of 0.839 for Lung Qi Deficiency vs health, cumulative R2Y of 0.965 and Q2 of 0.876 for Lung-Kidney Qi Deficiency vs health, and cumulative R2Y of 0.968 and Q2 of 0.889 for Lung-Spleen Qi Deficiency vs health. Finally, after filtering with multivariate analysis (VIP > 1 in PLSDA analysis) and univariate analysis (p <0.05 and fold change > 1.3 defined as statistically significant), and matching the changed features with public metabolites databases and reference standards in accurate MS and MS/MS, 1, 3, and 3 changed metabolites were annotated from these features as biomarker compounds for three TCM patterns, respectively (**Table 2**). In positive ion mode, we identified 1537 features and, similarly, the differences between COPD with three TCM patterns and healthy subjects were also apparent in the PLS-DA 3D scatterplot (**Figure 2A**), with a cumulative R2Y of 0.994 and Q2 of 0.847 for Lung Qi Deficiency vs health, cumulative R2Y of 0.942 and Q2 of 0.833 for Lung-Kidney Qi Deficiency vs health, and cumulative R2Y of 0.986 and Q2 of 0.909 for Lung-Spleen Qi Deficiency vs health. With the same criterion, 1, 2, and 3 features were annotated as biomarker compounds for three TCM patterns of COPD, respectively (**Table 2**).

**Table 2:** Statistical analysis of diagnostic biomarkers from metabolomics.

Name	*m/z*	Retention time (min)	Trend	Fold change	p-value	VIP
Lung Qi Deficiency VS health						
Cortisol	363.2165	9.36	↓	7.69E-01	3.60E-04	1.42
Palmitoleic acid	253.2169	20.09	↑	1.52E+00	1.16E-02	1.24
Lung-Kidney Qi Deficiency VS health						
Creatine	132.0768	0.72	↓	7.63E-01	5.32E-03	1.05
Bilirubin	585.2706	24.80	↓	7.30E-01	6.90E-07	2.04
Myristic acid	227.2014	19.72	↑	1.61E+00	4.42E-04	1.55
Palmitoleic acid	253.2169	20.09	↑	1.95E+00	5.99E-05	1.63
12,13-DiHOME	313.2385	16.96	↑	2.40E+00	4.89E-02	1.08
Lung-Spleen Qi Deficiency VS health						
Hypoxanthine	137.0458	0.83	↑	1.91E+00	9.43E-03	1.21
Bilirubin	585.2706	24.80	↓	6.60E-01	3.50E-05	1.44
Cortisol	363.2165	9.36	↓	7.20E-01	4.13E-04	1.69
3-Carboxy-4-methyl-5-propyl-2-furanpropionic acid (CMPF)	239.0924	10.30	↑	2.13E+00	1.23E-02	1.01
Palmitoleic acid	253.2171	20.09	↑	1.52E+00	1.18E-02	1.13
Glycochenodeoxycholic acid	448.3067	17.57	↑	2.42E+00	1.41E-02	1.08

**Figures 2B**, **C** display the Venn diagrams and pathway enrichment of the changed metabolites for the COPD patients with 3 TCM patterns vs healthy subjects. Among them, palmitoleic acid was included in the biomarker panels for all 3 TCM patterns. In addition, cortisol is the common biomarker for Lung Qi Deficiency and Lung-Spleen Qi Deficiency, and bilirubin is the common biomarker for Lung-Kidney Qi Deficiency and Lung-Spleen Qi Deficiency. At the same time, some metabolites can be used as characteristic biomarkers to distinguish different TCM patterns. Myristic acid, creatine, and 12,13-DiHOME, for example, can be used to differentiate Lung-Kidney Qi Deficiency from other patterns, and hypoxanthine 3-Carboxy-4-methyl-5 -propyl-2-furanpropionic acid (CMPF) and glycochenodeoxycholic acid can be used to differentiate Lung-Spleen Qi Deficiency. In metabolomics, no characteristic biomarker for Lung Qi Deficiency was found. In general, the metabolite biomarkers were mostly included in the pathways of porphyrin and chlorophyll metabolism and arginine and proline metabolism.

### Changes of lipid class/subclass between COPD patients and healthy subjects

In order to fully characterize the metabolic difference of COPD patients in different TCM patterns, a high-coverage pseudo-targeted lipidomics approach was applied on the basis of metabolomics, which was described in detail in our previous research (13). **Figure 1C** shows the PCA scatterplot of QC samples and test samples, and **Figure 1F** shows the reproducibility of commonly detected lipids in pseudotargeted lipidomics. We can see that QC samples (QC samples are analyzed after every 8 injections) were gathered together on the scatterplot, proving that the experiment was stable during analysis.

**Figure 3:**
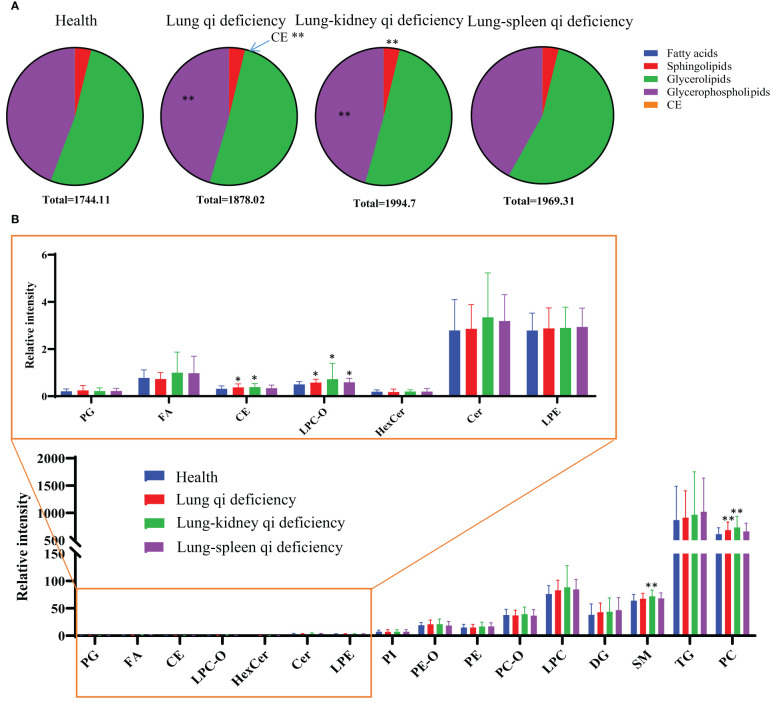
Lipid distribution pie charts **(A)** and relative quantity of each lipid subclass **(B)** in healthy subjects, COPD of different TCM patterns. Relative quantities (peak area ratio vs corresponding subclass IS at the same concentration) of all identified lipid species were summed per lipid class for each individual sample. *p < 0.05, **p < 0.01 vs healthy subjects. CE, cholesterol ester; Cer, ceramide; DG, diacylglycerol; FA, fatty acid; Health, healthy subjects; HexCer, hexaglycosylceramide; LPC, lyso-phosphatidylcholine; LPC-O, lysophosphatidylcholine with alkyl substituents; LPE, lyso-phosphatidylethanolamine; PC, phosphatidylcholine; PC-O, alkyl- and alkenyl-substituted phosphatidylcholine; PE, phosphatidylethanolamine; PE-O, alkyl- and alkenyl-substituted phosphatidylethanolamine; PG, phosphatidylglycerol; PI, phosphatidylinositol; PS, phosphatidylserine; SM, sphingomyelin; TG, triacylglycerol.

To investigate the changes in the lipid pool, the total content of each lipid class/subclass was determined for each sample; for each lipid, this was calculated as its peak area divided by that of the IS of the same subclass (*C*_perIS_) (**Figure 3**). As the ISs for each subclass had the same concentration, the relative contents of different lipid classes/subclasses were comparable. There is no statistically significant difference between the *C*_perIS_ of total lipids of healthy subjects and patients with COPD of different subtypes. Compared with healthy subjects, Lung Qi Deficiency and Lung-Kidney Qi Deficiency showed higher levels of glycerophospholipids, and Lung Qi Deficiency showed higher levels of cholesterol ester (CE) (**Figure 3A**). For each subclass of lipids, serum sphingolipids (including ceramide (Cer) and sphingomyelin (SM)) showed no difference in COPD of Lung Qi Deficiency and Lung-Spleen Qi Deficiency compared to healthy subjects, only Lung-Kidney Qi Deficiency showed significantly increased SMs (p<=0.01)(**Figure 3B**), the Cers level was higher in Lung-Kidney Qi Deficiency patients but not statistically significant. Glycerophospholipids showed a more significant difference between healthy subjects and COPD patients with different TCM patterns. Among glycerophospholipids, all TCM patterns showed a higher level of ether-containing glycerophospholipids (LPC-Os) (**Figure 3B**), and the patients with Lung Qi Deficiency and Lung-Kidney Qi Deficiency showed a higher level of phosphatidylcholine (PC).

### Cross-comparisons of patients with COPD of different TCM patterns by lipidomics

**Figure 4:**
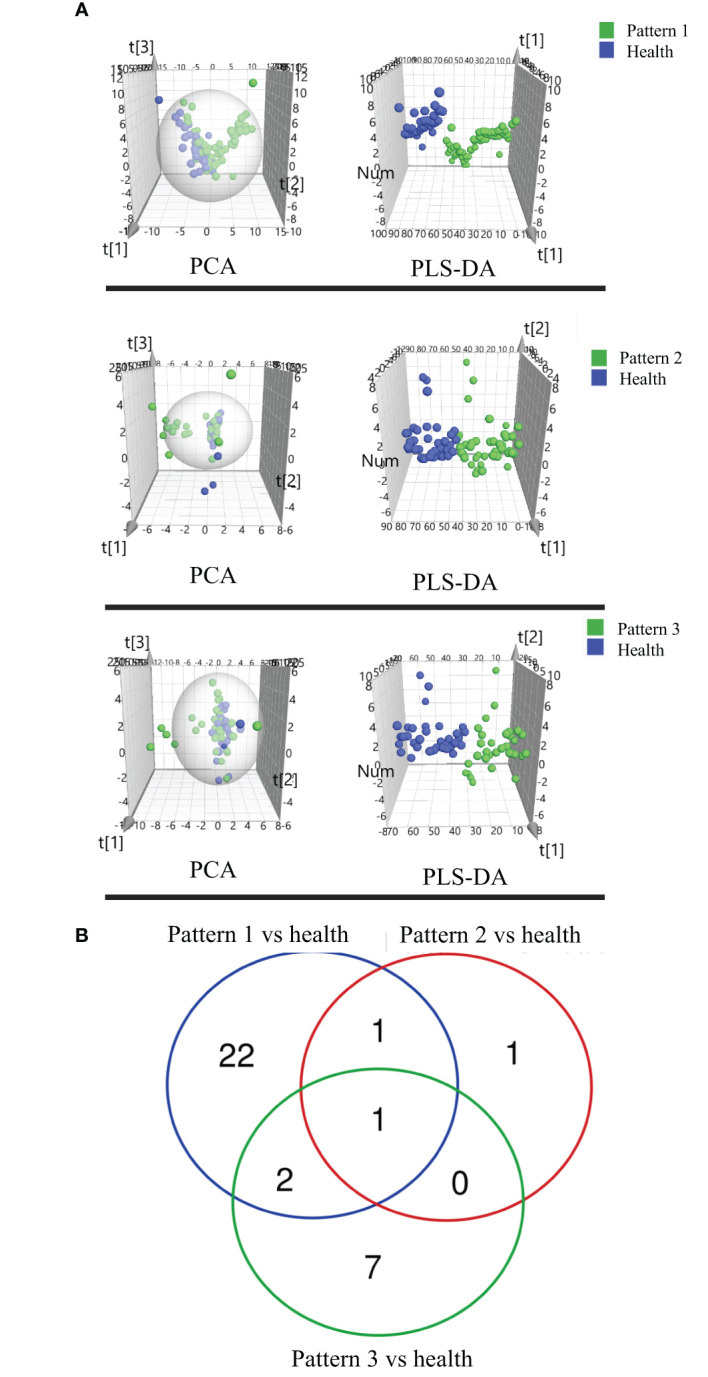
**(A)** Score plots of PCA and PLSDA based on the cross-comparison of Lung Qi Deficiency (Pattern 1) vs health, Lung-Kidney Qi Deficiency (Pattern 2) vs health, and Lung-Spleen Qi Deficiency (Pattern 3) vs health and **(B)** Venn diagrams of the changed lipids for COPD patients with 3 TCM patterns vs healthy subjects by lipidomic analysis.

We compared the COPD patients with different TCM patterns to healthy subjects and identified 150 lipids (107 lipids for Lung Qi Deficiency vs Health, 28 lipids for Lung-Kidney Qi Deficiency vs Health, and 59 lipids for Lung-Spleen Qi Deficiency vs Health) that were significantly altered (p<0.05, fold change >1.2) (19). Then a PLS-DA model was used to characterize metabolic disturbances. Lipids with VIP values of >1.0 were considered candidate differential lipids (19). As a result, 43, 9, and 23, respectively, were identified as differential lipids for Lung Qi Deficiency, Lung-Kidney Qi Deficiency, and Lung-Spleen Qi Deficiency. The differences between COPD with three TCM patterns and health were apparent in the PCA and PLS-DA 3D scatterplot (**Figure 4A**), with a cumulative R2Y of 0.748 and Q2 of 0.536 for Lung Qi Deficiency vs health, a cumulative R2Y of 0.664 and Q2 of 0.570 for Lung-Kidney Qi Deficiency vs health, and a cumulative R2Y of 0.764 and Q2 of 0.692 for Lung-Spleen Qi Deficiency vs health in PLSDA.

For the differential diagnosis of COPD of different TCM patterns using lipidomics, we used the criteria VIP >1.2, adjusted p-value <0.05, and fold change >1.2 as the filters. As a result, there were 26 lipid biomarkers that could distinguish between COPD of Lung Qi Deficiency and healthy subjects, as well as three for Lung-Kidney Qi Deficiency vs health, and 10 for Lung-Spleen Qi Deficiency vs health (**Table 3**). A Venn diagram of the differential lipids is shown in **Figure 4B**. We can also see that some lipid markers of TCM patterns of COPD are common to health, such as PC 16:0_18:3-1/PC 16:1_18:2, but there were more markers that differed among these groups that could potentially be used for differential diagnosis. In total, 22 lipids can be used as character biomarkers to distinguish Lung Qi Deficiency from the other two TCM patterns of COPD; similarly, there are one and seven lipids that can be used to distinguish Lung-Kidney Qi Deficiency and Lung-Spleen Qi Deficiency, respectively.

**Table 3:** Statistical analysis of diagnostic biomarkers from lipidomics.

ID	Name	*m/z*	Retention time (min)	Trend	Fold Change	p-value	VIP
Lung Qi Deficiency vs health							
230	PE-O 18:1p_22:4-2	778.5746	10.79	↓	5.40E-01	3.29E-03	2.37
194	PC-O 16:1e_22:6	790.5707	8.86	↑	1.23E+00	5.03E-03	1.86
124	LPC-O 18:1p-5	506.3608	2.41	↑	1.62E+00	3.60E-03	1.73
308	TG 16:0_18:0_18:0	881.5204	5.85	↑	1.32E+00	3.29E-03	1.65
204	PE 16:0_18:3/PE 16:1_18:2	714.5060	8.19	↓	4.76E-01	6.97E-03	1.65
200	PC-O 18:2e_22:6/PC-O 20:3_20:5e	816.5877	9.11	↑	1.57E+00	3.60E-03	1.56
365	PG 18:0_18:2/PG 18:1_18:1-2	804.5898	10.62	↑	2.38E+00	3.60E-03	1.55
151	PC 16:0_18:3-1/PC 16:1_18:2	756.5539	7.98	↓	6.11E-01	0.00E+00	1.47
156	PC 16:0_22:4	810.6023	9.40	↑	5.07E+00	0.00E+00	1.46
121	LPC-O 18:0p-2	508.3761	2.69	↑	2.27E+00	2.32E-03	1.44
250	SM d18:1_18:2/SM d18:2_18:1	881.5204	5.85	↓	7.09E-01	3.29E-03	1.43
76	DG 18:2_22:5-1	684.5556	9.93	↑	2.88E+00	4.63E-03	1.43
14	Cer d16:1_20:0/Cer d17:0_19:1/Cer d18:1_18:0	566.5495	10.75	↑	1.28E+00	3.29E-03	1.42
115	LPC 24:0-2	608.4653	5.88	↑	3.00E+00	2.99E-03	1.42
198	PC-O 18:0p_22:6	818.6031	9.99	↑	1.36E+00	5.03E-03	1.35
179	PC 18:2_20:4	806.5714	8.06	↑	1.78E+00	0.00E+00	1.34
110	LPC 22:5-1	570.3552	1.55	↑	2.69E+00	5.03E-03	1.33
174	PC 18:1_22:5/PC 20:2_20:4	834.5992	9.08	↑	3.32E+00	2.72E-14	1.33
386	PI 18:2_20:4-1	804.5898	10.62	↓	5.14E-01	4.63E-03	1.32
181	PC 19:0_20:4	824.6154	10.29	↓	8.30E-01	5.03E-03	1.30
66	DG 18:1_20:5-1	658.5397	9.88	↑	1.20E+00	8.74E-08	1.30
183	PC 20:4_22:6	854.5687	7.49	↓	6.68E-01	3.60E-03	1.29
130	LPC-O 20:1p-2	534.3911	2.33	↓	8.13E-01	3.60E-03	1.28
63	DG 18:1_19:0	654.6028	12.23	↑	1.42E+00	5.03E-03	1.26
51	DG 16:0_22:5/DG 18:1_20:4	660.5549	10.65	↑	1.32E+00	1.18E-03	1.21
176	PC 18:2_18:2	782.5712	8.16	↑	1.73E+00	0.00E+00	1.21
Lung-Kidney Qi Deficiency vs health							
151	PC 16:0_18:3-1/PC 16:1_18:2	756.5539	7.98	↓	7.25E-01	0.00E+00	1.92
156	PC 16:0_22:4	494.3241	1.56	↑	5.36E+00	0.00E+00	1.69
179	PC 18:2_20:4	494.3241	1.56	↑	2.02E+00	0.00E+00	1.34
Lung-Spleen Qi Deficiency vs health							
252	SM d18:1_24:1	881.5204	5.85	↑	1.39E+00	0.00E+00	1.92
151	PC 16:0_18:3-1/PC 16:1_18:2	756.5539	7.98	↓	8.20E-01	0.00E+00	1.59
247	SM d17:1_24:1/SM d18:1_23:1/SM d18:2_23:0	881.5204	5.85	↓	8.32E-01	0.00E+00	1.47
89	LPC 16:1	494.3241	1.56	↑	1.38E+00	4.43E-03	1.31
277	TG 14:0_16:0_20:5/TG 14:0_18:1_18:4/TG 14:0_18:2_18:3/TG 14:1_16:0_20:4/TG 14:1_18:1_18:3/TG 14:1_18:2_18:2/TG 16:0_16:1_18:4	881.5204	5.85	↑	1.21E+00	0.00E+00	1.28
51	DG 16:0_22:5/DG 18:1_20:4	660.5549	10.65	↑	1.22E+00	4.20E-04	1.25
229	PE-O 18:1p_18:2	726.5427	9.73	↓	7.99E-01	2.90E-02	1.24
156	PC 16:0_22:4	810.6023	9.40	↑	2.87E+00	0.00E+00	1.24
327	TG 16:1_16:1_20:4	881.5204	5.85	↑	1.23E+00	0.00E+00	1.23
242	SM d16:1_24:2/SM d18:1_22:2/SM d18:2_22:1	881.5204	5.85	↑	1.25E+00	3.12E-04	1.23

Details of the lipid identification and this lipid library seeing previous work (9).

### Differential diagnosis TCM patterns of COPD based on metabolic biomarkers

Accurate diagnosis of TCM pattern is critical for deciding the appropriate therapeutic strategy for patients and personalized COPD treatment. For the differential diagnosis of COPD using metabolomics and lipidomics-based biomarkers, receiver operating characteristic (ROC) analysis based on logistic regression was used to evaluate the accuracy of the biomarkers based on metabolomics, lipidomics, and both combined.

**Figure 5:**
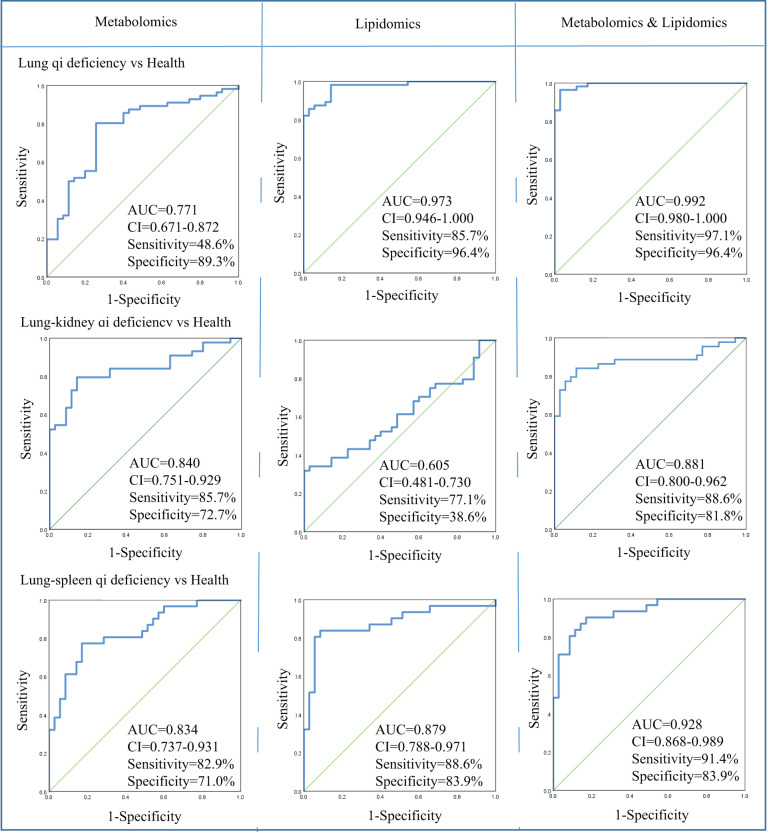
Diagnostic utility of differential metabolites as determined by receiver operating characteristic (ROC) curve analysis by metabolomics and lipidomics. AUC, area under the receiver operating characteristic curve; CI, confidence interval; Health, healthy subjects; VIP, variable importance in projection.

In metabolomics analysis, there were two biomarkers that could distinguish between COPD of Lung Qi Deficiency and health subjects, five for Lung-Kidney Qi Deficiency vs health, and six for Lung-Spleen Qi Deficiency vs health (**Table 2**). When metabolomic biomarkers were used alone as diagnostic criteria, the ROC analysis was conducted based on logistic regression of three biomarker panels, the area under the ROC curve (AUC), sensitivity, and specificity were 0.771, 48.6%, and 89.3%, respectively, for Lung Qi Deficiency vs healthy subjects; 0.834, 82.9%, and 71.0%, respectively, for Lung-Kidney Qi Deficiency vs healthy subjects; and 0.840, 85.7%, and 72.7%, respectively, for Lung-Spleen Qi Deficiency vs healthy subjects (**Figure 5**). When lipidomic biomarkers are used alone as diagnostic criteria, the AUC, sensitivity, and specificity were 0.973, 85.7%, and 96.4%, respectively, for Lung Qi Deficiency vs healthy subjects; 0.879, 88.6%, and 83.9%, respectively, for Lung-Kidney Qi Deficiency vs healthy subjects; and 0.605, 77.1%, and 38.6%, respectively, for Lung-Spleen Qi Deficiency vs healthy subjects (**Figure 5**). Combining the results of metabolomics and lipidomics, the prediction efficiency of the model is significantly increased in ROC analysis. The AUC, sensitivity, and specificity were 0.992, 97.1%, and 96.4%, respectively, for Lung Qi Deficiency vs healthy subjects; 0.928, 91.4%, and 83.9%, respectively, for Lung-Kidney Qi Deficiency vs healthy subjects; and 0.881, 88.6%, and 81.8%, respectively, for Lung-Spleen Qi Deficiency vs healthy subjects (**Figure 5**).

**Figure 6:**
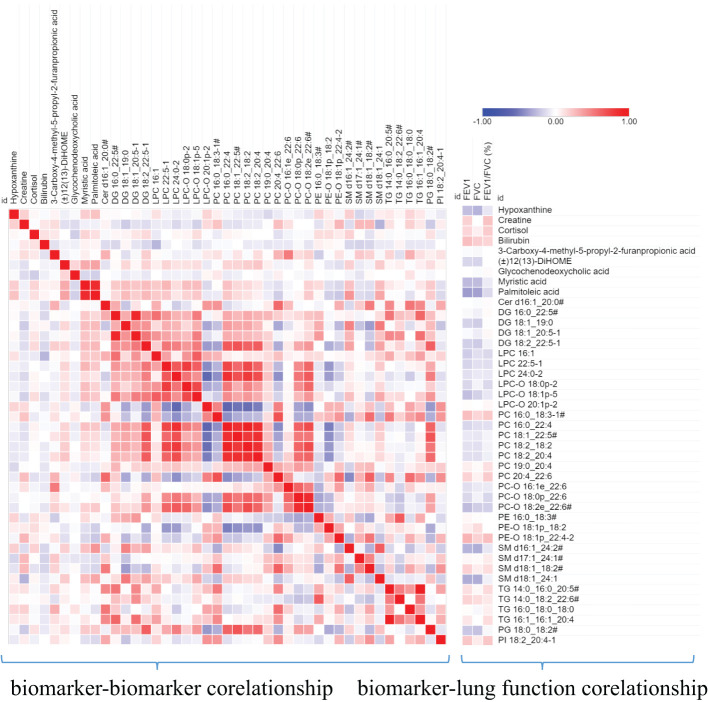
A heat map shows the correlation between metabolite biomarker and biomarker and lung function parameters. The degree of correlation is indicated by a gradient of red (positive) and blue (negative). # the metabolites identified with isomers, which seeing **Table 3**.

Furthermore, we also analyzed the correlation between these biomarkers and the correlation between these biomarkers and the lung function parameters. As shown in **Figure 6**, Bilirubin and PE-O 18:1p_22:4 is positively correlated with lung function parameters, while SM d16:1_24:2/SM d18:1_22:2/SM d18:2_22:1, SM d18:1_ 24:1, Palmitoleic acid, and myristic acid are negatively correlated with lung function parameters. Among biomarkers, we can see that there is a significant positive correlation between the same lipid subclasses, such as PCs and PC-Os, which indicates the consistency of lipid class or subclass changes.

## Discussion

**Figure 7:**
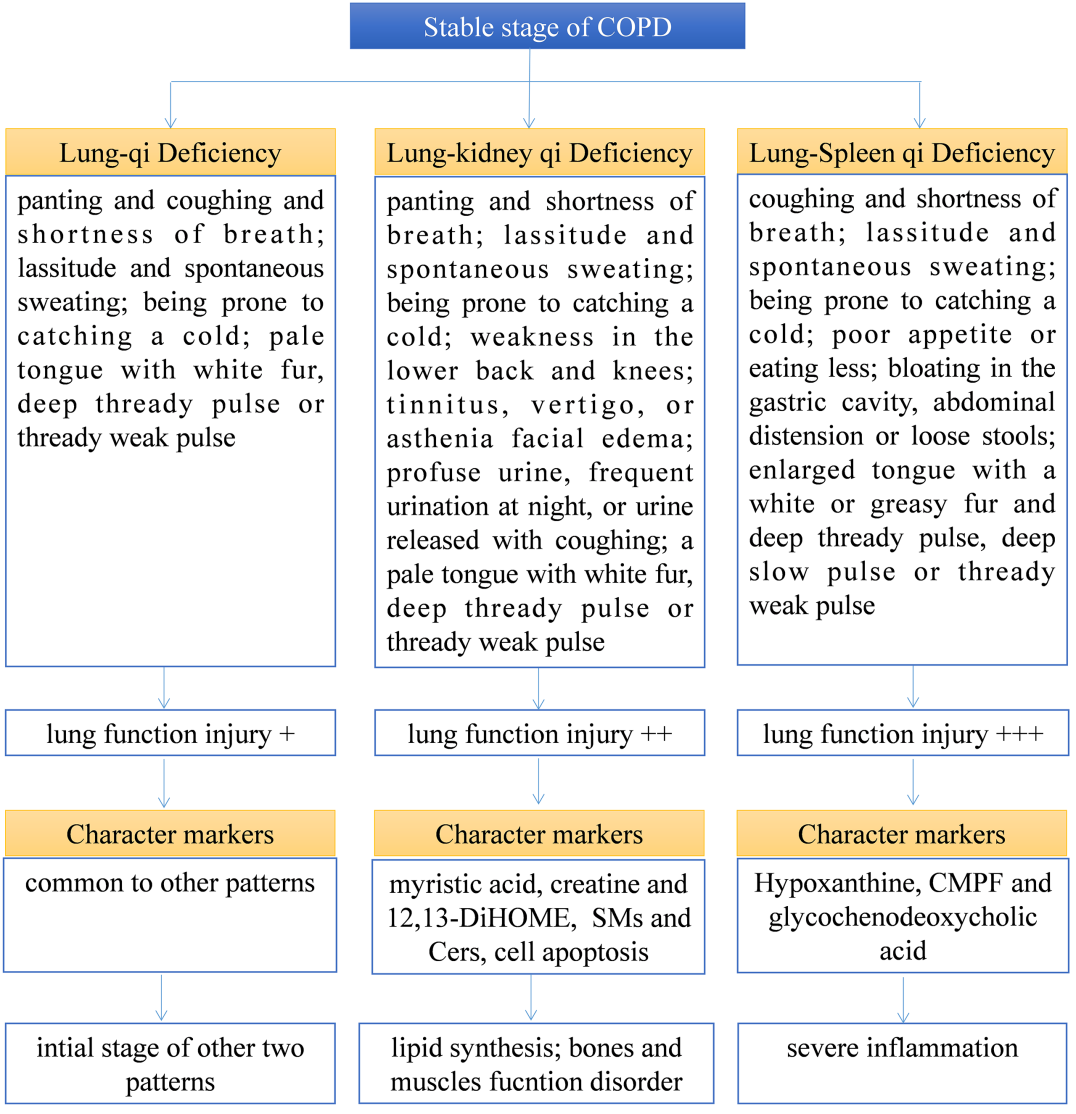
Summary of the clinical diagnosis, lung function, metabolic marker, and biological characteristics of different TCM patterns in COPD.

TCM pattern differentiation is the cornerstone for TCM practitioners to understand diseases and guide individualized clinical medication. However, TCM pattern differentiation has a certain degree of complexity, ambiguity, and subjectivity (20). Firstly, the syndrome description of TCM patterns is based on human patients, which is difficult to transfer to animal models for simulation or experiment. Therefore, this experiment aims to find biomarkers with different TCM patterns from 171 serum samples of patients with COPD that are strictly screened clinically, which can indicate more accurate characteristics of TCM patterns. Secondly, to find the biological bases of different TCM patterns clinically, we still need to classify the different TCM patterns of COPD through TCM practitioners first, and it is necessary to ensure the accuracy and repeatability of this process. First of all, this work depends on the standard of diagnosis and treatment of COPD in TCM jointly constructed by hospitals in China. The standard specifies the classification of different TCM patterns of COPD and the corresponding medication suggestions so as to help TCM practitioners make an accurate diagnosis. At the same time, in order to further ensure accurate diagnosis, each subject was diagnosed by two researchers respectively, and the person in charge of the research unit finally determined whether there was any inconsistency. As shown in **Table 1**, there is no significant difference in the baseline parameters of patients with COPD of different TCM patterns, but there is a significant difference in lung function. Among them, lung function damage in Lung-Spleen Qi Deficiency is the most obvious, followed by Lung-kidney Qi Deficiency, and finally, Lung Qi Deficiency. This can be partly explained from the perspective of the TCM understanding of lung disease. In TCM theory, Lung Qi Deficiency is the initial stage of lung disease, on the other hand, there is a close correlation between various organs; the dysfunction of the lungs will affect other organs such as the spleen and kidneys, and the function damage in other organs will also feed back to further damage lung function. Among them, the “Spleen” in TCM is responsible for the transportation of food and produces the refined Qi of water and grain, which is the basis of Qi generation. Deficiency of Spleen Qi causes obstructions of water and dampness and leads to the build-up of dampness and turbidity the accumulation of dampness and phlegm in lung tissue damages lung function. Therefore, lung function is seriously weakened due to Lung-Spleen Qi Deficiency, while Lung Qi Deficiency causes the least damage to lung function. However, the influencing factors of lung function are very complex. Natural aging and other diseases are accompanied by the decline of lung function, such as FEV1; thus, normal elderly people are often misdiagnosed with COPD according to spirometry (21, 22). Therefore, the difference in lung function may be the result of different TCM patterns, but there is no evidence to distinguish TCM patterns according to lung function indicators. Some previous studies have shown that some metabolic biomarkers have been found to effectively characterize the two main TCM patterns of acute exacerbation of COPD, phlegm–heat congesting lung, and phlegm-damp amassing in the lung (13). In this experiment, we further combined metabolomics and lipidomics, two bioinformatics tools for clinical high-throughput screening to analyze the metabolic biological basis and diagnostic biomarkers of different TCM patterns of COPD. **Figure 7** summarizes the clinical diagnosis, lung function, metabolic markers, and biological characteristics of different TCM patterns in COPD.

Firstly, we found three panels of metabolic biomarkers based on metabolomics to diagnose different TCM patterns of COPD, and their AUC in ROC analysis was 0.771-0.840. As previously mentioned, the conventional metabolomics strategy can only detect polar or moderately polar metabolites due to their extraction and chromatographic analysis methods. Thus, lipidomics is often parallelly used to characterize the nonpolar lipids. In lipidomics, the non-polar lipid fraction was extracted from the serum using MTBE, then in the chromatographic analysis, the C8 reversed-phase chromatographic column was used as the stationary phase, and water-acetonitrile-isopropyl alcohol was used as the mobile phase so that the non-polar lipid could be better separated. As a supplement to metabolomics, it helps us find more diagnostic biomarkers. For the lipidomics strategy that we used here, all lipids in the serum detected by accurate *m/z* and MS/MS spectra were first identified, then semi-quantitative and statistical analyses were conducted on the samples based on this target lipid library to find the differential lipids. This pseudo-targeted lipidomics approach can reduce identification errors caused by the non-targeted method and avoid the interference of background signal on statistical parameters. As expected, when combining lipid biomarkers with previous metabolomics biomarkers as diagnostic criteria, its AUC in ROC analysis increased to 0.881-0.992. It can be seen that the combination of lipidomics and metabolomics can increase the prediction accuracy of metabolic biomarkers by improving the coverage of the analysis.

Based on the above results, we can not only identify a group of metabolic biomarkers for clinical diagnosis but also understand the pathogenesis of COPD from the biological processes involved using these biomarkers. In metabolomics analysis, there is no marker compound to differentiate Lung Qi Deficiency from the other TCM patterns (the only two biomarkers, cortisol and palmitoleic acid, are common to two other patterns). This may be because Lung-Kidney Qi Deficiency and Lung-Spleen Qi Deficiency are the further development of the Lung Qi Deficiency in pathology; thus, both have the characteristics of Lung Qi Deficiency. However, besides the characteristics of Lung Qi Deficiency, patients with Lung-Kidney Qi Deficiency also have Kidney Qi Deficiency syndrome; thus, myristic acid, creatine, and 12,13-DiHOME can be used to distinguish patients with Lung-Kidney Qi Deficiency. Myristic acid is a principal substrate for protein myristoylation and a potential peroxisomal β-oxidation product (23). The increase in myristic acid can reflect the increase in lipid synthesis, which is consistent with the increase in the overall level of lipids in COPD, especially in Lung-Kidney Qi Deficiency patients (**Figure 3A**). In addition, the level of creatine is significantly lower in patients with Lung-Kidney Qi Deficiency. In TCM, Kidney Qi Deficiency is characterized by weakness in the lower back and knees, that is, the function of bones and muscles is decreased, which may be related to the decline of creatine. 12,13-DiHOME is an oxylipin; the literature indicates that 12,13-diHOME can improve metabolic health, and the action of this molecule appears to favor the absorption of fatty acids by brown adipose tissue and stimulate the browning process in white adipose tissue (24). In patients with Lung-Kidney Qi Deficiency, 12,13-DiHOME may be negative feedback of the increase of lipid synthesis. However, there are few studies on this lipid function, and further experimental verification is needed. Hypoxanthine, 3-Carboxy-4-methyl-5-propyl-2-furanpropionic acid (CMPF) and glycochenodeoxycholic acid has been proven suitable for distinguishing patients with Lung-Spleen Qi Deficiency. Hypoxanthine is an important purine metabolite, and it causes hyperuricemia due to excessive synthesis to uric acid. Hypoxanthine is also associated with inflammation (25). Thus, the increase in hypoxanthine may indicate some pathological processes related to COPD inflammation. CMPF is a kind of uremic toxin (26); however, some studies have shown that it may also be used as a biomarker of type 2 diabetes (27). Because the concept of the spleen in TCM is different from that in modern medicine, it refers to the whole digestive system organ. So, the pancreatic islet, the organ of diabetes, belongs to the “Spleen” in TCM, and CMPF may be a marker related to pancreatic islet function to be studied. Glycochenodeoxycholic acid is the predominant human dihydroxy bile salt under cholestatic conditions and is a likely candidate for bile acid-mediated hepatocellular injury during cholestasis due to its direct cytotoxic effects on hepatocytes (28). There are few studies on this metabolite, and the relationship between it and the pathological process of COPD is still unclear.

In the lipidomics analysis, we used a pseudo-targeted lipidomics approach; therefore, we can both understand the difference in lipid class/subclass and the difference in individual lipids between different groups. Among them, sphingolipids and glycerophospholipids are the lipid classes with the largest difference between various TCM patterns and healthy subjects. Sphingolipids are primarily found in cell membranes but also in biofluids such as plasma and serum and are involved in diverse biological processes, including cell death, proliferation, differentiation, autophagy, senescence, migration, and efferocytosis (29). Sphingolipid metabolism was shown to be dysregulated by smoking and COPD (30–32). Sphingolipid synthesis was enhanced due to lung injury, especially Cers, which enhanced pulmonary vascular cell apoptosis (33) and decreased clearance of apoptotic cells by alveolar macrophages (34). Compared to healthy subjects and other TCM patterns of COPD, Lung-Kidney Qi Deficiency showed increased SMs and Cers, which indicates that Lung-Kidney Qi Deficiency may be distinguished by enhanced pulmonary vascular cell apoptosis, although this requires validation in additional studies. In addition, the increase of LPC-Os may be a characteristic of all TCM patterns of COPD compared with healthy subjects. This is somewhat inconsistent with previous research, which states that the enhanced oxidative stress in COPD development may lead to reduced plasmalogen (35); thus, the reason of increased LPC-Os in COPD patients showed in this work is also worthy further study.

As previously mentioned, the biological basis of TCM patterns is commonly induced by low throughput molecular biological methods from different levels. This study attempts to explain the biological basis of TCM patterns at the metabolite level because the final results of changes in transcription and protein levels are difficult to infer due to complex biological signal interactions. As the final product of all biochemical reactions, the differences in metabolites can better reflect the results of physiological and pathological changes. Therefore, metabolomics is more suitable for the preliminary screening of the biological basis of TCM patterns and the discovery of biomarkers. However, metabolomics commonly only reflects pathological results but not the mechanisms of pathological changes or drug effects. Therefore, in the follow-up work, it will be beneficial to comprehensively analyze the results of this work combined with transcriptome and proteomics. By studying the differences in mRNA and protein levels in blood samples of patients with different TCM patterns to confirm the target of dialectical treatment of COPD, and by studying the differences in target mRNA and protein in blood samples of patients before and after receiving dialectical treatment, as well as studying the correlation between drug regulated targets and changes in clinical indicators, we can find the regulatory targets of the corresponding Chinese medicine in dialectical treatment to completely analyze the biological network of TCM pattern differentiation treatment of COPD.

## Conclusion

COPD is a complex disease, and dialectical treatment of TCM has been proven to be an important research direction for treating COPD. However, its diagnosis and treatment are mainly based on conventional TCM theory. This experiment combines metabolomics and lipidomics, aiming to study its biological mechanism from the metabolic level. The results indicate that Lung-Spleen Qi Deficiency showed the most serious lung function damage, followed by Lung-kidney Qi Deficiency and Lung-Qi Deficiency. At the metabolic level, Lung-Kidney Qi Deficiency is characterized by increased myristic acid (related to lipid synthesis), SMs and Cers (related to cell apoptosis), and reduced creatine (related to smooth muscle function). Lung-Spleen Qi Deficiency is characterized by increased hypoxanthine (related to inflammation)., However, the metabolic disturbances of Lung Qi Deficiency, as the early stage of COPD, do not have characteristics compared to the other two TCM patterns. In addition, one of the bottlenecks of dialectical treatment is the lack of quantitative standards. Biomarker screening based on multi-omics can provide some reference for the classification of these “TCM subtypes”. In this experiment, three panels of biomarkers found by metabolomics and lipidomics were used for the clinical characterization of three main TCM patterns of COPD. In future research, it is suggested that these biomarkers, or a combination of biomarkers with clinical dialectics, be used to establish a systematic TCM pattern classification strategy. The limitation of this experiment is that the sample size is still relatively small, which may have a significant impact due to individual differences, and further multicenter clinical experiments are needed to verify it. At the same time, further research is needed to reveal the etiology and pathogenesis mechanism of these metabolic disturbances.

## Data availability statement

The original contributions presented in the study are included in the article/**Supplementary Material**. Further inquiries can be directed to the corresponding author.

## Ethics statement

The studies involving human participants were reviewed and approved by The ethics committee of The First Affiliated Hospital of Henan University of Chinese Medicine. (Ethical approval number 2019HL-016-02). The patients/participants provided their written informed consent to participate in this study.

## Author contributions

JL: Conceptualization; Validation; Writing - Review & Editing. XL: Investigation; Formal analysis; Methodology; Writing - Original Draft. YS: Investigation; Sample collection. YX: Investigation; Validation; Writing - Review & Editing. JY: Investigation; Sample collection. AZ: Data collection. YD: Investigation. JW: Investigation; Formal analysis. All authors contributed to the article and approved the submitted version.

## Glossary

**Table:**

3D	3-dimensional
AUC	area under the receiver operating characteristic curve
BMI	body mass index
CE	cholesterol ester
Cer	ceramide
COPD	chronic obstructive pulmonary disease
DG	diacylglycerol
FA	fatty acid
Hex2Cer	dihexaglycosylceramide
FEV1	Forced expiratory volume in one second
FVC	forced vital capacity
HexCer	hexaglycosylceramide
IS	internal standard
LC-MS	liquid chromatography–mass spectrometry
LPC	lyso-phosphatidylcholine
LPC-O	lyso-phosphatidylcholine with alkyl substituents
LPE	lyso-phosphatidylethanolamine
LPE-O	lyso-phosphatidylethanolamine with alkyl substituents
MS/MS	tandem mass spectrometry
MTBE	methyl tert-butyl ether
PC	phosphatidylcholine
PC-O	alkyl- and alkenyl-substituted phosphatidylcholine
PE	phosphatidylethanolamine
PE-O	alkyl- and alkenyl-substituted phosphatidylethanolamine
PG	phosphatidylglycerol
PI	phosphatidylinositol
PLS-DA	projection to latent structures discriminant analysis
PS	phosphatidylserine
QC	quality control
ROC	receiver operating characteristic
RSD	relative standard deviation
SCOPD	stable chronic obstructive pulmonary disease
SM	sphingomyelin
TCM	traditional Chinese medicine
TG	triacylglycerol
tR	retention time
UHPLC-Q-orbitrap-MS	ultra-high performance liquid chromatography–quadrupole–orbitrap–mass spectrometry
VIP	variable importance in projection
