# Supplementary material for: Differentiation in TCM patterns of chronic obstructive pulmonary disease by comprehensive metabolomic and lipidomic characterization
Source: Front Immunol. 2023 Jul 10;14:1208480. doi: 10.3389/fimmu.2023.1208480 (PMC10363632; doi:10.3389/fimmu.2023.1208480)
Supplement: Supplementary file 1 [file Table_1.docx]

Table S1 The concentration of 9 internal standards in stock solution and extraction solution.

| Internal standards | Stock solution  (mg/mL) | Extraction solution  (μg/mL) |
| --- | --- | --- |
| Tryptophan-d5 | 1.08 | 1.08 |
| Valine-d8 | 1.08 | 1.08 |
| Phenylalanine-d5 | 1.03 | 1.03 |
| Palmitic acid-d3 | 0.96 | 0.96 |
| Cholic acid-d4 | 1.25 | 1.25 |
| Stearic acid-d3 | 1.11 | 1.11 |
| Carnitine C8:0-d3 | 0.85 | 0.085 |
| Carnitine C16:0-d3 | 0.94 | 0.094 |
| LPC 19:0 | 10 | 0.50 |
